# Leaf unfolding of Tibetan alpine meadows captures the arrival of monsoon rainfall

**DOI:** 10.1038/srep20985

**Published:** 2016-02-09

**Authors:** Ruicheng Li, Tianxiang Luo, Thomas Mölg, Jingxue Zhao, Xiang Li, Xiaoyong Cui, Mingyuan Du, Yanhong Tang

**Affiliations:** 1 University of Chinese Academy of Sciences, Beijing 100049, China; 2 Key Laboratory of Alpine Ecology and Biodiversity, Institute of Tibetan Plateau Research, Chinese Academy of Sciences, Beijing 100101, China; 3 Climate System Research Group, Institute of Geography, Friedrich-Alexander-University Erlangen-Nürnberg (FAU), Erlangen 91058, Germany; 4 National Institute for Agro-Environmental Sciences, Tsukuba 305-8604, Japan; 5 National Institute for Environmental Studies, Tsukuba 305-8506, Japan; 6 CAS Center for Excellence in Tibetan Plateau Earth Sciences, Beijing 100101, China

## Abstract

The alpine meadow on the Tibetan Plateau is the highest and largest pasture in the world, and its formation and distribution are mainly controlled by Indian summer monsoon effects. However, little is known about how monsoon-related cues may trigger spring phenology of the vast alpine vegetation. Based on the 7-year observations with fenced and transplanted experiments across lower to upper limits of *Kobresia* meadows in the central plateau (4400–5200 m), we found that leaf unfolding dates of dominant sedge and grass species synchronized with monsoon onset, regardless of air temperature. We also found similar patterns in a 22-year data set from the northeast plateau. In the monsoon-related cues for leaf unfolding, the arrival of monsoon rainfall is crucial, while seasonal air temperatures are already continuously above 0 °C. In contrast, the early-emerging cushion species generally leafed out earlier in warmer years regardless of precipitation. Our data provide evidence that leaf unfolding of dominant species in the alpine meadows senses the arrival of monsoon-season rainfall. These findings also provide a basis for interpreting the spatially variable greening responses to warming detected in the world’s highest pasture, and suggest a phenological strategy for avoiding damages of pre-monsoon drought and frost to alpine plants.

Under global warming in the last 100 years, the trend of precipitation varies greatly with timescales and regions^1^. Alpine grasslands are one of the most vulnerable ecosystems to warming and reduced water availability in the world^2^. Identifying the correct environmental cues that initiate plant growth is the key to predicting how species and ecosystems respond to climate change^3^. While a great number of previous studies have found temperature and photoperiod cues for spring phenology of alpine plants^2^,^4^, the phenological response to precipitation change has been less studied despite its important role in shaping species distribution and ecosystem function^3^,^5^. It is still difficult to understand how spring phenology of alpine grasslands responds to warming because of variable precipitation change trends and limited knowledge about the complex evolutionary and plastic responses of plant life cycles^2^,^3^,^4^,^5^,^6^. On the Tibetan Plateau (TP), the climate characterized by Indian monsoon in summer (ISM) and the westerlies in winter has contributed to the formation and distribution of the highest and largest alpine grasslands in the world^7^,^8^. In response to the rapid warming over the TP during recent decades^9^, contrasting trends in satellite-derived green-up dates^10^,^11^,^12^ and insignificant trends in long-term observed leaf-out dates^13^,^14^ were recently reported. To complicate matters, there are some indications that ISM is weakening and the westerlies are strengthening since the 1970s, resulting in decreased precipitation in the Himalayas and southern TP but increased precipitation in northwest and northeast parts^15^. Tree-ring records of alpine treelines indicate a great increase of pre-monsoon droughts in recent decades^16^. To the best of our knowledge, few observed data have examined to what extent monsoon-related cues may trigger spring phenology of alpine vegetation, and how the cues can influence the greening response to air temperature.

The uplifted TP exerts a strong dynamic and thermal forcing on the regional atmospheric circulation, which affects the formation of ISM^8^,^17^. It has been suggested that the genera of *Kobresia* (sedge species) and *Androsace* (cushion species) originated in the Himalaya-Hengduan Mountains when the TP extensively uplifted in the early Miocene, and then differentiated and extended northward and westward^18^,^19^. The large-scale colonization of cushion-like *Androsace* species on the TP is thought to occur in the late Holocene^18^. Today, the alpine *Kobresia* meadow (3200–5600 m a.s.l) is the largest pasture with a long history of grazing on the TP^20^, in which *Kobresia pygmaea* is the most widespread and dominant species, and *Androsace tapete* (cushion-forming perennial herb, endemic to TP) is a widespread and keystone species serving as an ecosystem engineer^21^. The fan-shaped distribution pattern of the *Kobresia* meadow is mainly controlled by the ISM moisture^7^ (Fig. 1) through the atmospheric water transports along the high mountain valleys in eastern Himalayas^22^. Recent observations further indicate that the plant growth and distribution are mainly limited by water availability^23^,^24^. Phenology is a key component of fitness and can evolve relatively rapidly^25^. The monsoon-westerly interaction and the long history of grazing may have selected genotypes of sedges and grasses that respond better to pre-monsoon droughts, in which the timing of leaf unfolding may be a key adaptive trait in shaping species distribution^25^. Since the typical ISM circulation is established during late May and June^26^ when the TP air temperature is generally above 0 °C, leaf unfolding of the monsoon-adapted dominant sedge and grass species may have evolved to sense the arrival of monsoon rainfall to protect them from pre-monsoon drought and frost damage^27^. On the other hand, the cold-adapted cushion species may begin growth soon after snow melt in early spring as previously suggested^2^,^27^ because the compact cushion-shaped architecture can create a unique microhabitat with moderate temperature and water availability^28^,^29^. In this study, we test the hypothesis that leaf unfolding dates of dominant sedge and grass species synchronize with monsoon onset when seasonal air temperatures are already continuously above 0 °C, contrasting to the early-emerging cushion species that generally leaf out earlier in warmer years regardless of precipitation.

To test the hypothesis, we used 7-year observations (2007–2013) for leaf unfolding (LU) of three representative species (*K. pygmaea* sedge at 4400–5200 m; *Stipa capillacea* grass at 4400–4650 m; *A. tapete* cushion at 4400–5200 m) at 7 altitudes in Damxung of Tibet. We also used meteorological observations at each of the 7 altitudes (from lower to upper limits of the *Kobresia* meadow), which is within the reach of ISM influence^26^,^30^. Given the short-term observations in Damxung, the green-up dates at each of the 7 altitudes during 2001–2013 were calculated from the time-series data of Normalized Difference Vegetation Index (NDVI) from the Moderate Resolution Imaging Spectroradiometer (MODIS, 250 m × 250 m). We tested if the observed LU dates of dominant species and satellite-derived green-up dates (if they compared well to the ground data) were correlated with: 1) the onset date of a 5-days moving-average daily precipitation threshold when daily mean temperature was continuously above 0 °C for five consecutive days (referred to as rainfall onset), 2) the mean temperature and precipitation of 30 or 60 days before a multi-year mean LU date (sedge/grass, T_-30d_ and Pr_-30d_; cushion, T_-60d_ and Pr_-60d_) and the winter mean temperature (T_winter_), and 3) atmospheric circulation indices during the ISM onset period from 25 May to 24 June^26^, including the date of establishment of the large-scale ISM circulation and the mean central-TP wind speed in the upper troposphere (300 hPa). This 300-hPa wind speed can serve as a parameter of the monsoon-westerly interaction over the central Tibetan Plateau (west of 95°E) since lower 300-hPa wind speed implies stronger ISM influence and local convection and therefore more local rainfall^26^. We further tested the generality of our findings using the long-term observations of LU dates (1989–2010) for *K. pygmaea*^13^ and other two common grass species of *Festuca ovina* and *Elymus nutans*^14^ in Qumalai and Henan stations of southern Qinghai. These locations are at the margin of the typical ISM influence where the climate is characterized by an early onset of precipitation in April to May, possibly as a result of combined moisture imports from the westerlies and ISM^22^,^30^. We examined if the long-term LU dates of sedge and grass species in southern Qinghai also synchronize with the rainy season onset.

## Results

### Monsoon-related cues for leaf unfolding of sedge and grass species

LU dates of the three representative species typically varied little with altitude at altitudes below 4950 m but were significantly delayed at 5100–5200 m (Fig. 2a). LU dates of *A. tapete* were 30–40 days earlier than those of other two species. In particular, LU dates of *K. pygmaea* and *S. capillacea* converged to the same period of ISM onset (Fig. 2a). Below 4950 m, LU dates of *K. pygmaea* and *S. capillacea* were in phase with rainfall onset dates during 2007–2013 (Fig. 2b and Supplementary Fig. S1a), both were positively correlated (R = 0.86–0.92, P < 0.05). The interannual variation of LU dates in populations of *K. pygmaea* at 4400–4800 m was well compared with that of 300-hPa wind speed (R = 0.84, P < 0.05; Fig. 2b). At community level, satellite-derived green-up dates during 2001–2013, which were well correlated with observed LU dates of *K. pygmaea* below 4950 m during 2007–2013 (R = 0.90, P < 0.05; but not at higher altitudes, P > 0.05), also indicated clear correlations with rainfall onset (R = 0.74, P < 0.01) and the 300-hPa wind speed (R = 0.68, P < 0.01) (based on the data in Fig. 2b).

Similar patterns were also found in the long-term LU data (1989–2010) in Qumalai and Henan for *K. pygmaea* and two common grass species *F. ovina* and *E. nutans*, in which the observed LU dates showed insignificant trends in the past 22 years (P > 0.10) but were typically coincided with rainfall onset dates (sedge, Fig. 2c; grass, Supplementary Fig. S1b; R = 0.65–0.92, P < 0.001).

### Responses of leaf unfolding to changes in temperature and precipitation

The early-emerging cushion species generally advanced LU with increasing early-spring temperature (T_-60d_, Fig. 3a) regardless of precipitation (Pr_-60d_, Fig. 3b). In contrast, the sedge and grass species below 4950 m typically advanced LU with increasing pre-monsoon precipitation (Pr_-30d_, Fig. 3d,f) but exhibited no response or a delayed response to increasing temperature (T_-30d_, Fig. 3c,e). The same results were found in the transplants across the three representative species (insets in Fig. 3). For the populations of *K. pygmaea* at and above its distribution center (4950–5200 m), however, LU dates varied little with Pr_-30d_ and even turned to decrease with increasing T_-30d_ at the upper limit (5200 m, with a threshold of T_-30d_ < 5 °C and Pr_-30d_ > 30 mm indicated by the grey-shaded vertical columns in Fig. 3e,f). The site-specific patterns were not observed in their transplants from 5200 m to 5100 m and from 4950 m to 4800 m (insets in Fig. 3e,f).

In general, LU dates of the three representative species and their sensitivity to temperature and precipitation did not differ with experimental treatments of grazed (unfenced) vs ungrazed (fenced) and transplanted downwards vs transplanted control at the same altitude (Table 1, Supplementary Tables S1–S2).

Similar patterns were also found in the long-term observed LU data (1989–2010) of *K. pygmae* in Qumalai and Henan (Fig. 4). While a general increase in T_-30d_ was observed between 1980 and 2013 (insets in Fig. 4a,c), the LU dates varied little with T_-30d_ (Fig. 4a,c) but decreased with increasing Pr_-30d_, especially for the years with Pr_-30d_ < 30 mm (Fig. 4b,d). There was a reduced correlation of LU vs Pr_-30d_ for the years with Pr_-30d_ > 30 mm (Fig. 4b,d).

Across all the species in Damxung, Qumalai and Henan, the observed LU dates at the same altitude exhibited no response or an advanced response to higher winter temperature (T_winter_) (Supplementary Fig. S3).

## Discussion

**Figure 1:**
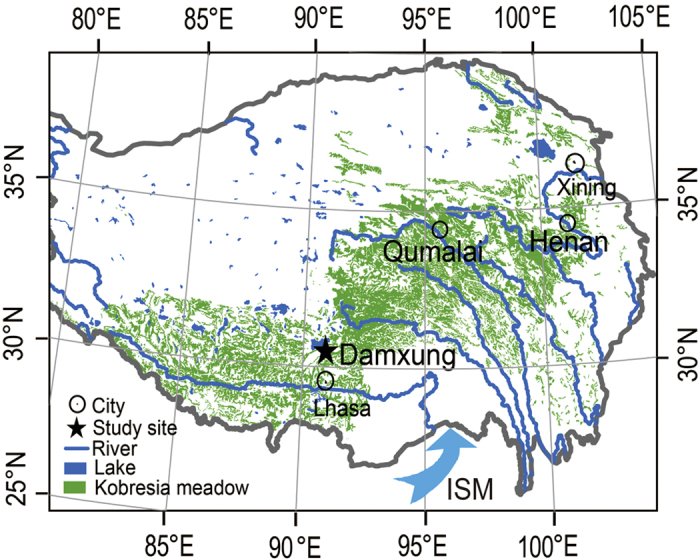
Map of alpine *Kobresia* meadows, ISM path and study sites, which was generated using ArcGIS 10.3 for Desktop based on the vector data of Tibetan *Kobresia* meadows digitized from Vegetation Atlas of China (1:1000000)^38^. The vector data are available free online at http://www.geodata.cn/Portal/metadata/viewMetadata.jsp?id=100101-48.

**Figure 2:**
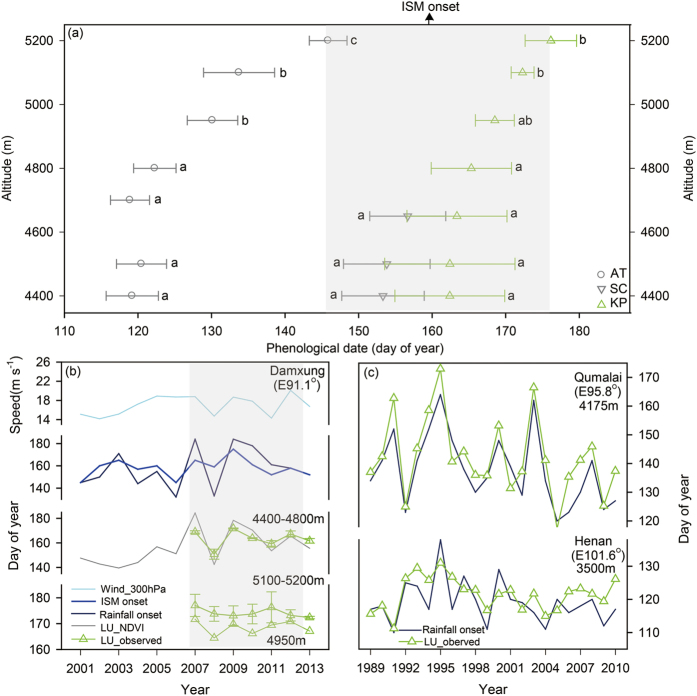
Leaf unfolding (LU) of three representative species varied with altitude and ISM onset. (**a**) Altitudinal patterns in mean LU dates for *Androsace tapete* (AT, open cycles), *Stipa capillacea* (SC, open invert-triangles), and *Kobresia pygmae* (KG, open triangles) in Damxung; the grey-shaded duration highlights the synchronization of LU with the ISM onset period (25 May to 24 June); different letters along altitude indicate significant differences in mean LU dates of 2007–2013 between altitudes at P < 0.05. (**b)** Annual variations in *Kobresia*’s LU dates of Damxung, ISM onset dates, rainfall onset dates, and 300-hPa wind speed in the ISM onset period during 2001–2013; the grey-shaded period is for our observed years. (**c**) Annual variations in *Kobresia*’s LU dates of Qumalai and Henan and related rainfall onset dates during 1989–2010.

**Figure 3:**
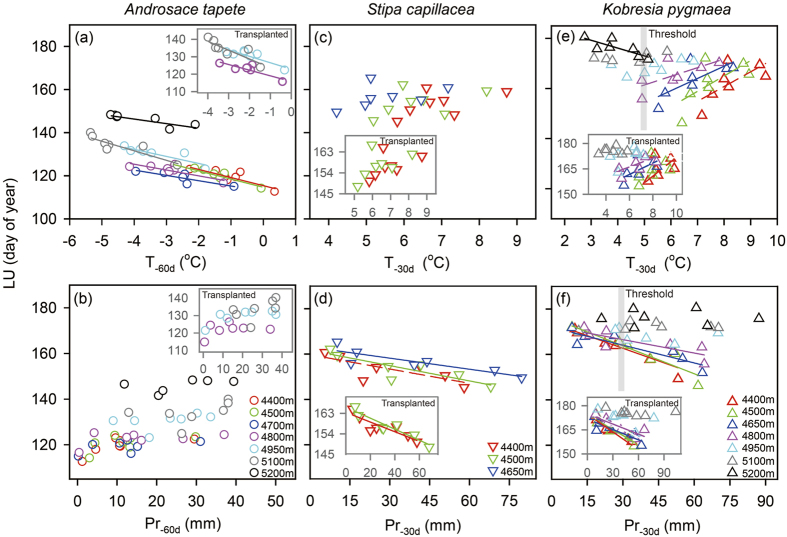
Relationships of leaf unfolding (LU) to mean temperature (T_-60d_, T_-30d_) and precipitation (Pr_-60d_, Pr_-30d_) of 60/30 days before mean LU dates for (**a, b**) *Androsace tapete*, (**c, d**) *Stipa capillacea*, and (**e, f**) *Kobresia pygmae* across altitudes in Damxung during 2007–2013. (I**nset**) Similar patterns were found in the transplants from a high altitude to a low altitude. (**e,f**) The grey-shaded vertical columns indicated the threshold of T_-30d_ < 5 °C and Pr_-30d_ > 30 mm for the specific pattern at the upper limit of *K. pygmae*. Solid trend lines are statistically significant at P < 0.05, and dash trend lines are for weak correlations at P < 0.10. Statistical differences in relationships of LU vs climatic factors among experimental treatments are found in Table 1.

For alpine ecosystems in semi-arid regions, plant phenology would be an adaptation to avoid environmental stress of cold and drought^3^,^27^. Our 7-year observations indicate that altitudinal LU dates of dominant sedge and grass species synchronized with monsoon onset when seasonal air temperatures were already continuously above 0 °C (Fig. 3 and Table 1). Similar patterns were also found in the northeastern distribution areas (Qumalai and Henan) of the *Kobresia* meadows based on the 22-year data set available in the literature (Fig. 2c, Supplementary Fig. S1b). Within species of *K. pygmaea*, the mean LU dates decreased from Damxung (91°05´E, DOY 163 ± 5, mean ± SD) to Qumalai (95°47´E, DOY 142 ± 14) and Henan (101°36´E, DOY 122 ± 5) with an advance rate of approximately 4 days per longitude degree eastward, which compares well to the earlier onset of the rainy season with increasing longitude (Damxung, DOY 157 ± 18; Qumalai, DOY 138 ± 12; Henan, DOY 119 ± 6) (Fig. 2b,c) and is consistent with the spatial patterns in satellite-derived green-up dates^10^,^11^. In Qumalai and Henan, where the ISM moisture from the south is imported more easily due to the large valleys^22^ (Fig. 1), the earlier LU onset triggered by a low rainfall threshold (>2 mm) is related to the earlier onset of precipitation in April to May when the daily mean precipitation is relatively low^30^, though the precise mechanisms cannot be detected. In previous satellite studies, chilling requirement^11^,^31^ or spring precipitation^10^ is speculated to be an important regulator of greening response to the warming on the TP. Warming in winter may delay the fulfillment of chilling requirements and thus lead to later onset of spring phenology^11^. Our data suggest that there would be no chilling requirement in Tibetan alpine meadows because the LU dates exhibited no response or an advanced response to higher winter temperature (Supplementary Fig. S3). Because of large spatiotemporal variations in the arrival of monsoon rainfall, which mainly depends on the onset of the ISM circulation and its intensity as well as on complex effects of topography^22^,^30^, variable greening change trends of the *Kobresia* meadows are expected. For example, the notably delayed trend in satellite-derived green-up dates found in the Himalayas and southern TP^10^ is most likely caused by the delayed arrival of monsoon rainfall and decreased pre-monsoon precipitation (Fig. 2b) regardless of air temperature variability. Our findings suggest a phenological strategy for avoiding damages of pre-monsoon drought and frost to alpine plants, which might also explain why there is a longitudinal decrease in temperature sensitivity of spring vegetation phenology from most coastal areas to inlands over the Northern Hemisphere^32^.

**Figure 4:**
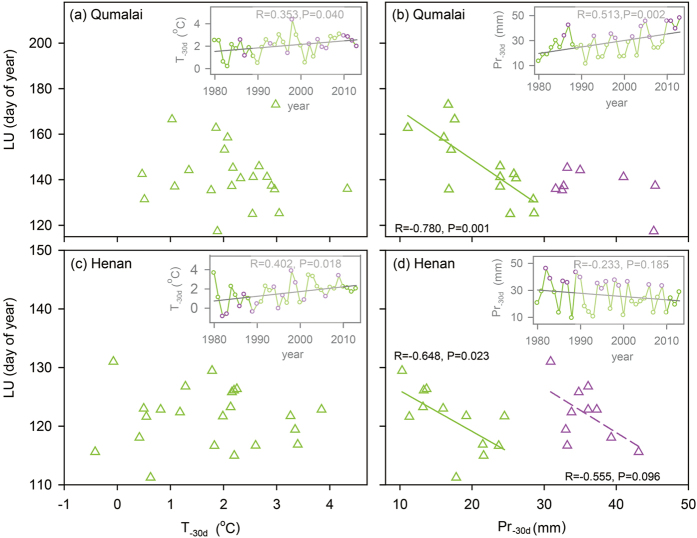
Relationships of *Kobresia pygmae*’s LU dates to mean temperature (T_-30d_) and precipitation (Pr_-30d_) 30 days before mean LU date during 1989–2010 in (**a,b**) Qumalai and (**c,d**) Henan. (Inset) The annual change trends of (**a**,**c**) T_-30d_ and (**b**,**d**) Pr_-30d_ during 1980–2013 based on time-series data of daily mean temperature and precipitation from the stations of China Meteorological Administration. The data were separated into two groups with Pr_-30d_ < 30 mm (green color) and Pr_-30d_ > 30 mm (red color) (both T_-30d_ were less than 5 °C across years and sites), according to the threshold for the specific pattern at the upper limit of *K. pygmae* in Damxung (Fig. 3e,f). Solid trend lines are statistically significant at P < 0.05, and dash trend lines are for weak correlations at P < 0.10.

Our data further indicated that the rainfall onset was positively correlated with ISM onset during 2001–2013 (R = 0.73, P < 0.01, based on the data in Fig. 2b), and the Pr_-30d_ was negatively correlated with ISM onset and rainfall onset (Supplementary Fig. S2). This suggests that earlier ISM onset might occur in concert with increased pre-monsoon precipitation (Pr_-30d_), which would explain why the dominant sedge and grass species typically advanced LU with increasing Pr_-30d_ (Figs 3d,f and 4b,d). The monsoon onset in general implies a weakening of the zonal flow in the upper troposphere, which reduces the vertical wind shear and enables formation of deeper convective clouds than before^33^. Therefore, if the ISM circulation is established early, it is likely that a weakening tendency of the zonal flow is well evident in May and precedes the ISM onset, which would also increase convection and precipitation in the pre-monsoon season^26^.

It should be noted that variable LU correlations with T_-30d_ and Pr_-30d_ were observed in the areas near the upper or northern limits of the *Kobresia* meadows (Figs 3e,f and 4, Table 1) where there is a large snowfall contribution to the total precipitation in the pre-monsoon season when the nighttime precipitation is generally high^30^,^31^. Because the solid-phase water has no effect on plant growth, the snowfall may dampen the liquid-phase monsoon rainfall cues for leaf unfolding when the temperature is low and the precipitation is high (T_-30d_ < 5 °C and Pr_-30d_ > 30 mm). This may explain why there was a reduced correlation of LU vs Pr_-30d_ at the higher altitudes (4950–5200 m) in Damxung (Fig. 3f) and for the years with Pr_-30d_ > 30 mm in Qumalai and Henan (Fig. 4b,d), and why the negative correlation of LU vs T_-30d_ at 5200 m and the insignificant correlation of LU vs Pr_-30d_ above 4950 m were not observed in their transplants from 5200 m to 5100 m and from 4950 m to 4800 m, respectively (insets in Fig. 3e,f). The early-season snowfall effects may also explain why the observed LU dates of *K. pygmaea* above 4800 m were not significantly correlated with the rainfall onset and the 300-hPa wind speed (Fig. 2b). On the other hand, the positive correlation between LU and T_-30d_ in *K. pygmaea* below 4950 m (Fig. 3e), which is consistent with the data of a short-term warming experiment nearby our study area^34^, may not be a causal relationship because such a correlation was not found for the same sedge species in Qumalai and Henan (Fig. 4a,c). Increased precipitation may lead to stronger surface cooling and lower surface temperatures, which may result in an indirect correlation of LU and T_-30d_. Based on the long-term climate data (1989–2013), there was a negative correlation between T_-30d_ and Pr_-30d_ in Damxung (R = − 0.51, P = 0.01) but not in Qumalai (R = 0.12, P = 0.57) and Henan (R = −0.14, P = 0.52), consistent with the positive correlation of LU and T_-30d_ in Damxung (4400–4800 m) but the insignificant correlation of LU and T_-30d_ in Qumalai and Henan.

Our data support the recent suggestion that the early-emerging species’ phenology should be more sensitive to abiotic forces^3^. As expected, the early-emerging cushion species generally leafed out earlier in warmer years regardless of precipitation (Fig. 3a,b), suggesting that early-emerging cushion plants may inevitably suffer pre-monsoon drought stress^23^ and would be most vulnerable to the weakening ISM and climatic warming. This may reduce the biodiversity and stability of the alpine ecosystems because cushion species often provide suitable habitat for other species in the harsh environments^21^,^28^. Given the greening response of dominant sedge and grass species to the timing of ISM onset, the productivity of Tibetan alpine meadows may be mainly altered by reduced water availability as a result of the weakening ISM and/or warming-enhanced pre-monsoon droughts^23^,^24^, compared to previous predictions for alpine ecosystems in Europe and North America based on the roles of photoperiod and snowmelt timing^2^,^4^.

In conclusion, our data provide evidence that leaf unfolding of dominant sedge and grass species responds directly to the arrival of monsoon rainfall, contrasting to the early-emerging cushion species that generally leaf out earlier in warmer years regardless of precipitation. These findings also provide a basis for interpreting the spatially variable greening responses to warming detected in the world’s highest pasture, and further suggest that the monsoon-related evolutionary history would play an important role in characterizing plant phenology and its temperature sensitivity. This study is the first step toward identifying an ecologically meaningful, measurable precipitation cue that initiates alpine plant growth.

## Methods

### Observations and experiments in Damxung of Tibet

On the central TP, Nyaiqentanglha Mountains lie in the zonal ecotone between alpine *Stipa* steppe and alpine *Kobresia* meadow, and are characterized by a semi-arid climate. Along the south-facing slope (30°30′–30°32′N, 91°03′E), vegetation types changed from the steppe-meadow dominated by *S. capillacea* at 4300–4650 m, to the typical meadow dominated by *K. pygmaea* at 4700–5210 m^23^,^24^. Other coexisting species mainly included *A. tapete*, *Arenaria lancangensis*, *Potentilla nivea*, *Carex atrofusca* etc. The main soil types along the slopes included the alpine meadow soil at ≥4600 m and the prairie meadow soil at <4600 m^7^. In August 2005, 7 HOBO weather stations (Onset Inc., Bourne, MA) were set up at 4400 m, 4500 m, 4650 m, 4800 m, 4950 m, 5100 m, and 5200 m along the slope. Air temperature (1.5 m aboveground) and precipitation were recorded at 30-minute intervals^23^,^24^.

In alpine grasslands, the removal of aboveground biomass by livestocks may modify soil temperature and moisture and snow melting, which might result in local effects on spring phenology. To clarify the uncertainty of local effects induced by grazing, LU dates were observed across fenced and unfenced treatments during 2007–2013. In May 2006, seven 20 × 20 m fenced plots were set nearby the HOBO weather stations at each of the 7 altitudes. Within each fenced plot, we set 5 long-term monitoring quadrats (50 × 50 cm) at intervals of two meters along the contour line. Also, we set 5 unfenced quadrats along the contour line at a distance of two meters from the upper edge of the fenced plot. Additionally, 30 unfenced quadrats were randomly sampled at three lower altitudes (4430 m, 4500 m, 4700 m) where *A. tapete* was sparsely present. The vertices of each quadrat were marked by four yellow labels. From early April to early November during 2007–2013, we made photos for each quadrat with an Olympus digital camera (2048 × 1536 pixels) once every 3–4 days when the sky was generally clear at 11:00–14:00. With the yellow labels as a control, the green pixels for each clump of *A. tapete* and each 10 × 10-cm sub-quadrat of *K. pygmaea* and *S. capillacea* within a quadrat were determined by means of Adobe Photoshop Elements. Species-specific LU dates for a cushion clump or a sedge/grass sub-quadrat were defined as the date of the green-pixel fraction >10% following the criteria of China Meteorological Administration^35^. Species-mean LU dates for a quadrat were averaged from all the clumps or the sub-quadrats. At an altitude, species-mean LU dates were averaged from the 5 fenced (unfenced) quadrats.

To investigate if the LU sensitivity to temperature and precipitation may differ with altitudes because of local adaptations^2^,^3^,^4^,^5^,^6^, we conducted an altitudinal transplant experiment. At each of the 7 altitudes, we set ten 25 × 25 cm quadrats at intervals of two meters along the contour line in a fenced plot, in which five quadrats were transplanted downward to the next fenced plot at a lower altitude (TD) and other five quadrats remained for transplanted control (TC). In April 2007 before greening, the TD and TC quadrats were carefully dug out to a depth of 30 cm. While the TC quadrats were left *in situ*, the TD quadrats with soil clod were carefully removed from a high altitude to a low altitude (5200 m to 5100 m, 5100 m to 4950 m, 4950 m to 4800 m, 4800 m to 4650 m, 4650 m to 4500 m, 4500 m to 4400 m). The method of LU observations was the same as described above.

### Long-term LU data

To test the generality of our results from Damxung, we collected the literature data of long-term observed LU dates (1989–2010) for *K. pygmaea* in Qumalai and Henan by Wang *et al.*^13^ and other two common grass species (*Festuca ovina* in Qumalai, *Elymus nutans* in Henan) by Xu *et al.*^14^. The observation methods followed the criteria of the China Meteorological Administration^35^, in which LU date was defined as the date when >10% of located plant individuals leafed out or greened up.

Given the short-term time series for Damxung, the green-up dates at each of the 7 altitudes during 2001–2013 were calculated from the time-series 250 m × 250 m MODIS-NDVI data of the MOD13Q1 product (MODIS/Terra Vegetation Indices 16-Day L3 Global 250 m SIN Grid) using the relative threshold method (the green-up date is defined as the date when a vegetation index reaches 20% of its annual amplitude)^11^,^36^.

### Climate factors and atmospheric circulation indices

Long-term climate data of daily mean temperature and precipitation in Damxung, Qumalai and Henan stations during 1980–2013 were obtained from China Meteorological Administration (CMA, http://cdc.nmic.cn/home.do). The CMA station data and our data from the 7 HOBO weather stations were used for calculating climate factors of rainfall onset, T_winter_ (December to February), and T_-30d_ and Pr_-30d_ for sedge/grass species or T_-60d_ and Pr_-60d_ for cushion species. Rainfall onset was defined as the onset date of a 5-days moving-average daily precipitation threshold when daily mean temperature was continuously above 0 °C for five consecutive days. The precipitation threshold may differ with species and sites, possibly because of the geographic variations in monsoon-westerly interaction and rainfall intensity. By using different precipitation thresholds (ranging from 1 mm to 5 mm by a step of 0.5 mm) within a species and site, we calculated the correlation coefficients between LU and rainfall onset based on time series data of LU dates and related daily mean temperature and precipitation. The optimum precipitation threshold for a species and site was determined according to the highest correlation coefficient between LU and rainfall onset: >4.5 mm for sedge and grass in Damxung, >2 mm (>1.5 mm) for sedge (grass) in Qumalai and Hena. T_winter_ was selected because the warming in winter may delay the fulfillment of chilling requirements and thus lead to later onset of spring phenology^11^,^31^. To further explore ecologically meaningful, interannually comparable climate factors associated with the long-term change of LU, the mean temperature and precipitation of 30 or 60 days before a multi-year mean LU date (T_-30d_ and Pr_-30d_ or T_-60d_ and Pr_-30d_) were used in this study.

The onset date of the large-scale ISM circulation (ISM onset) and the central-TP mean wind speed at 300 hPa in the ISM onset period (300-hPa wind speed) were calculated from the NCEP/NCAR reanalysis^37^ using the method of Mölg *et al.*^31^. Accordingly, the typical ISM onset period is from 25 May to 24 June^26^. This is the time window in which the characteristic ISM circulation in a particular year is established.

### Data analysis

One-way ANOVA and the Tukey HSD test were applied to assess differences in mean LU dates of 2007–2013 among different altitudes and between experimental treatments at an altitude in Damxung. A simple linear model (y = a + bx) was used to determine the temporal trends in LU dates and climate factors as well as the relationships between LU dates and climate factors (ISM variables) and between climate factors and ISM variables. Analysis of covariance (ANCOVA) in a general linear model framework was used to test for differences in slopes of linear relationships between LU and climate factors among different altitudes or different experimental treatments (LU as a dependent variable, climate factor as a covariate, and altitude/treatment as a grouping variable).

All statistical analysis was performed using SPSS 20.0 for Windows, and all significant differences were taken at P < 0.05.

## Additional Information

**How to cite this article**: Li, R. *et al.* Leaf unfolding of Tibetan alpine meadows captures the arrival of monsoon rainfall. *Sci. Rep.* **6**, 20985; doi: 10.1038/srep20985 (2016).

## Supplementary Material

Supplementary Information

## Figures and Tables

**Table 1:** Slopes of linear relationships of leaf unfolding (LU) to mean temperature (T_-60d_ or T_-30d_) and precipitation (Pr_-60d_ or Pr_-30d_) for *Androsace tapete*, *Stipa capillacea* and *Kobresia pygmae* along altitudes during 2007–2013.

Species & Altitude	Temperature				Precipitation			
	UF	F	TD	TC	UF	BF	TD	TC
Androsace tapete								
4400 m	−3.7^*A^				0.26			
4500 m	−3.8^*A^				0.21			
4700 m	−2.5^*A^				0.14			
4800 m	−2.7^*Aa^	−2.7^*Aa^	−3.2^*Aa^	−2.9^*Aa^	0.10	0.10	0.10	0.10
4950 m	−3.3^*Aa^	−3.2^*Aa^	−2.2^*Aa^	−3.0^*Aa^	0.17	0.17	0.20	0.19
5100 m	−4.4^*Aa^	−4.2^*Aa^	−5.1^*Aa^	−4.4^*Aa^	0.24	0.23	0.34	0.33
5200 m	−2.1^*Aa^	−2.2^*Aa^			0.06	0.05		
Stipa capillacea								
4400 m	3.1	2.7	2.6	2.7	−0.20^*Aa^	−0.20^*Aa^	−0.20^*Aa^	−0.20^*Aa^
4500 m	2.5	3.1	3.0	3.1	−0.20^*Aa^	−0.23^*Aa^	−0.23^*Aa^	−0.23^*Aa^
4650 m	2.3	2.3			−0.16^*Aa^	−0.16^*Aa^	−0.16^*Aa^	−0.16^*Aa^
Kobresia pygmaea								
4400 m	5.9^*Aa^	5.5^*Aa^	5.5^Aa^	3.0^Aa^	−0.37^*Aa^	−0.37^*Aa^	−0.37^*Aa^	−0.37^*Aa^
4500 m	6.1^*Aa^	5.4^*Aa^	3.3	3.8	−0.41^*Aa^	−0.36^*Aa^	−0.31^*Aa^	−0.36^*Aa^
4650 m	4.9^*Aa^	4.3^*Aa^	3.4^Aa^	3.6 ^Aa^	−0.25^*Aa^	−0.21^*Aa^	−0.21^*Aa^	−0.21^*Aa^
4800 m	3.5^*Aa^	2.9^*Aa^	2.6^Aa^	3.0^Aa^	−0.20^*Aa^	−0.19^*Aa^	−0.21^*Aa^	−0.19^*Aa^
4950 m	1.4	1.0	2.1	1.6	−0.05	−0.06	−0.12	−0.06
5100 m	1.1	0.1	0.1	0.2	−0.04	−0.03	−0.03	−0.03
5200 m	−3.1^*Ba^	−1.5^*Ba^			−0.01	−0.02		

The asterisks (*) are for significant linear relationships between LU and climatic factors at P < 0.05. The slope differences were tested by analysis of covariance (ANCOVA). Different letters within a column and a row show significant differences in the slopes between altitudes (capital) and between treatments (lowercase) of unfenced (UF), fenced (F), transplanted downwards (TD) and transplanted control (TC) at P < 0.05, respectively.
