# Supplementary material for: Leaf unfolding of Tibetan alpine meadows captures the arrival of monsoon rainfall
Source: Sci Rep. 2016 Feb 9;6:20985. doi: 10.1038/srep20985 (PMC4809099; doi:10.1038/srep20985)
Supplement: Supplementary Information [file srep20985-s1.pdf]

Title: Leaf unfolding of Tibetan alpine meadows captures the arrival of monsoon rainfall

Running title: Leaf-out date synchronizes with monsoon onset

Ruicheng Li[1,2], Tianxiang Luo[2*], Thomas Mölg[3], Jingxue Zhao[2], Xiang Li[2], Xiaoyong Cui[1], Mingyuan Du[4], Yanhong Tang[5‡]

[1]University of Chinese Academy of Sciences, Beijing 100049, China

[2]Key Laboratory of Alpine Ecology and Biodiversity, Institute of Tibetan Plateau Research, Chinese Academy of Sciences, Beijing 100101, China

[3]Institute of Geography, University of Erlangen-Nuremberg, Erlangen 91058, Germany

[4]National Institute for Agro-Environmental Sciences, Tsukuba 305-8604, Japan

[5]National Institute for Environmental Studies, Tsukuba 305-8506, Japan

[‡]Current Address: Department of Urban and Environmental Science, Peking University, Beijing 100871, China

[*]**Corresponding author:**

Dr. Tianxiang Luo

Key Laboratory of Alpine Ecology and Biodiversity,

Institute of Tibetan Plateau Research, Chinese Academy of Sciences

Building 3, 16 Lincui Rd., Chaoyang District, Beijing 100101, China

Fax: +86 10 84097060

Email: luotx@itpcas.ac.cn

Text and display items: abstract 200 words, main text 2708 words, Methods 1187 words, 38 references, Figs 1-4, Table 1

Supplementary information: Appendix tables S1-S2 and figures S1-S3

Type of paper: Articles

Key words: alpine meadow, monsoon onset, phenology, precipitation, warming

Online supplementary information: Appendix tables S1-S2 and figures S1-S3

Table S1 One-way ANOVA of species-specific LU difference between experimental treatments of grazed (unfenced) vs ungrazed (fenced) at the same altitude in Damxung of Tibet during 2007-2013.

Species & Altitude	Sum of Squares		df		F	Sig.
	Between groups	Within groups	Between groups	Within groups		
Androsace tapete						
4800	4.07	735.79	1	68	0.376	0.542
4950	36.67	1777.34	1	68	1.054	0.308
5100	37.64	1369.07	1	68	1.870	0.176
5200	14.63	1237.33	1	68	0.804	0.373
Stipa capillacea						
4400	2.07	379.13	1	68	0.375	0.540
4500	2.64	500.40	1	68	0.359	0.551
4650	2.38	390.64	1	68	0.381	0.532
Kobresia pygmaea						
4400	12.35	1931.15	1	68	0.435	0.512
4500	5.36	1811.70	1	68	0.201	0.655
4650	22.30	1449.52	1	68	1.046	0.310
4800	16.18	1111.22	1	68	0.990	0.323
4950	0.07	368.34	1	68	0.012	0.904
5100	0.16	355.19	1	68	0.031	0.860
5200	0.08	388.92	1	68	0.013	0.908

Table S2 One-way ANOVA of species-specific LU difference between experimental treatments of transplanted downwards vs transplanted control at the same altitude in Damxung of Tibet during 2007-2013.

Species & Altitude	Sum of Squares Between groups	Sum of Squares Within groups	df Between groups	df Within groups	F	Sig.
Androsace tapete						
4800	5.26	735.81	1	68	0.486	0.488
4950	8.08	963.49	1	68	0.418	0.510
5100	34.29	1369.04	1	68	1.703	0.196
Stipa capillacea						
4400	2.08	380.13	1	68	0.376	0.540
4500	2.54	504.40	1	68	0.360	0.550
Kobresia pygmaea						
4400	15.12	1931.41	1	68	0.532	0.468
4500	7.23	1811.78	1	68	0.271	0.604
4650	26.00	1449.43	1	68	1.220	0.273
4800	19.28	1111.23	1	68	1.180	0.281
4950	0.125	578.37	1	68	0.015	0.901
5100	2.33	355.56	1	68	0.990	0.323

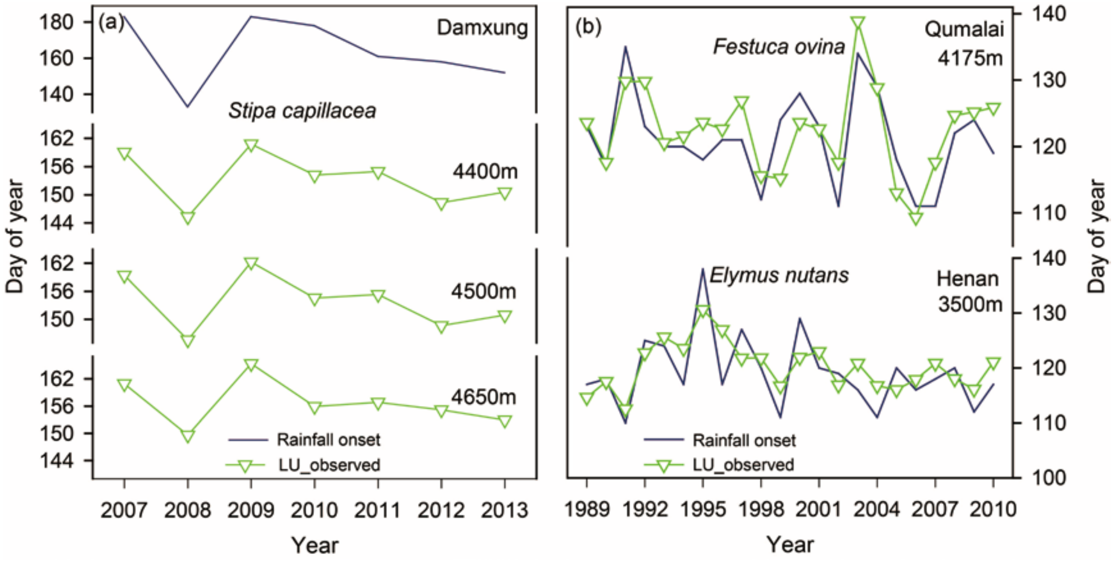

Fig. S1 Annual variations in rainy season onset (blue symbols and lines) and LU dates (green symbols and lines) across grass species: (**a**) *Stipa capillacea* in Damxung (2007-2013), (**b**) *Festuca ovina* in Qumalai and *Elymus nutans* in Henan (1989-2010). Observed LU dates of *F. ovina* and *E. nutans* were obtained from Xu *et al*. (2014).

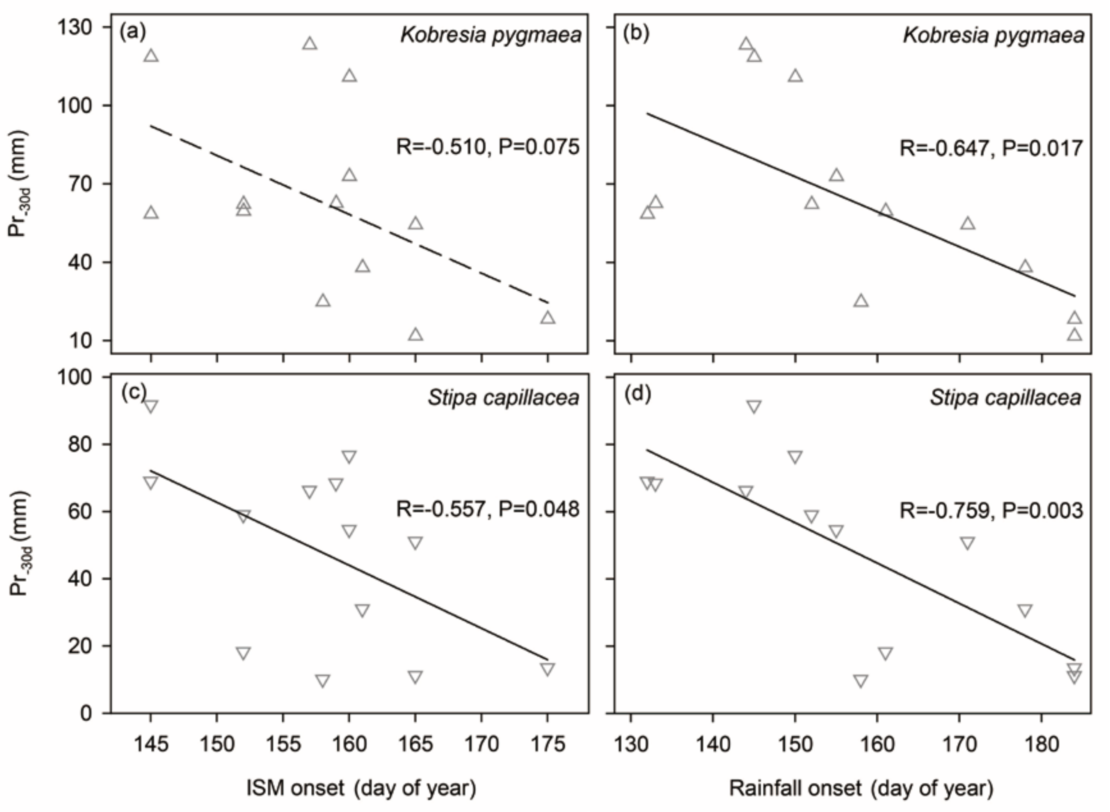

Fig. S2 Relationships of Pr_{-30d} to ISM onset and rainfall onset during 2001-2013 across sedge (a-b) and grass (c-d) species in Damxung. Pr_{-30d} was for the precipitation of 30 days before mean LU dates of *Kobresia pygmae* at 4400-4800 m (DOY 163±5, mean±SD) and of *Stipa capillacea* at 4400-4650 m (DOY 155±5), respectively.

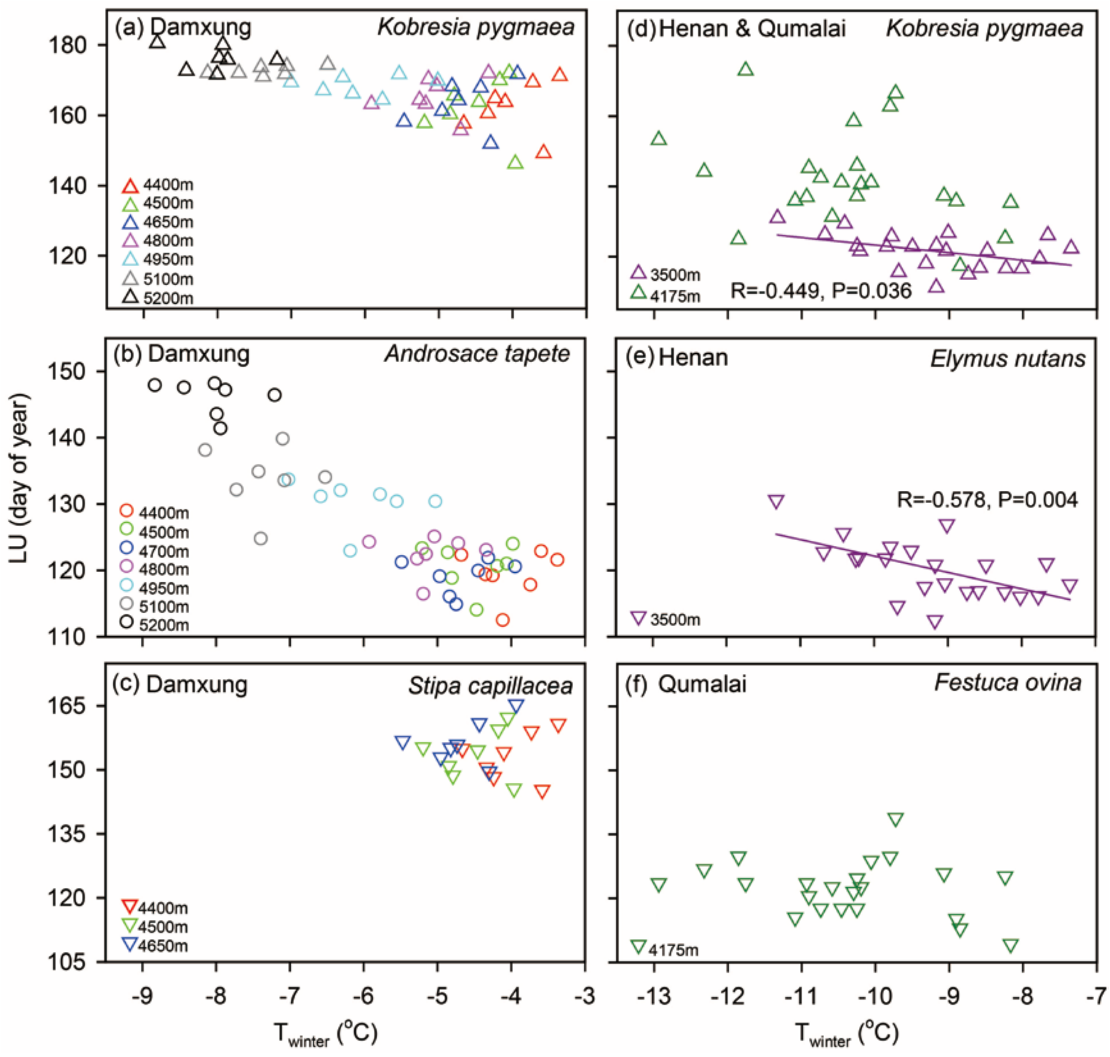

Fig. S3 Relationships between species-specific LU date and winter mean temperature (T_{winter}) at the same altitude in (**a–c**) Damxung during 2007-2013 and (**d-f**) Qumalai and Henan during 1989-2010. The observed LU dates in Qumalai and Henan were obtained from Wang *et al.* (2012) and Xu *et al.* (2014).
